# Quantitative Measurement and Evaluation of Red Blood Cell Aggregation in Normal Blood Based on a Modified Hanai Equation

**DOI:** 10.3390/s19051095

**Published:** 2019-03-04

**Authors:** Jianming Wen, Nen Wan, Huilu Bao, Jianping Li

**Affiliations:** The Institute of Precision Machinery and Smart Structures, College of Engineering, Zhejiang Normal University, Jinhua 321004, China; wjming@zjnu.cn (J.W.); wannen@zjnu.cn (N.W.); huilubao@163.com (H.B.)

**Keywords:** red blood cell, aggregation, blood, impedance, relaxation frequency

## Abstract

The aggregation of red blood cells (RBCs) in normal blood (non-coagulation) has been quantitatively measured by blood pulsatile flow based on multiple-frequency electrical impedance spectroscopy. The relaxation frequencies *f*_c_ under static and flowing conditions of blood pulsatile flow are utilized to evaluate the RBC aggregation quantitatively with the consideration of blood flow factors (RBC orientation, deformation, thickness of electrical double layer (EDL)). Both porcine blood and bovine blood are investigated in experiments, for the reason that porcine blood easily forms RBC aggregates, while bovine blood does not. The results show that the relaxation frequencies *f*_c_ of porcine blood and bovine blood present opposite performance, which indicates that the proposed relaxation frequency *f*_c_ is efficient to measure RBCs aggregation. Furthermore, the modified Hanai equation is proposed to quantitatively calculate the influence of RBCs aggregation on relaxation frequency *f*_c_. The study confirms the feasibility of a high speed, on-line RBC aggregation sensing method in extracorporeal circulation systems.

## 1. Introduction

Cardiovascular diseases (CVDs) have been reported to account for 31% of the total mortality all over the world, according to the World Health Organization (WHO) [1]. Patients with serious CVDs have microcirculatory disorders that are found to be strongly associated with the biophysical properties of blood [2]. Hence, the biophysical properties of blood, such as the viscosity [3,4,5], hematocrit [6,7], RBC deformability [8,9], erythrocyte sedimentation rate (ESR) [10,11], RBC aggregation [12,13,14] and other factors [15,16,17], are widely tested to diagnose the pathological or physiological disorders of CVDs. Among the biophysical properties of blood, RBC aggregation is the major cause of the non-Newton flow properties of whole blood, and it has been popularly utilized to diagnose the inflammatory diseases in clinic, since the level of aggregation rises enormously in association with diabetes [18], sepsis [19], myocardial ischaemia [20] and so on. Up to now, various researchers have paid attention to the measurement of RBC aggregation, and some novel techniques based on laser backscattering [21,22], microscopic counter [23,24], ultrasound backscattering [25,26,27] and electrical impedance spectroscopy [28,29] have been developed and described.

Compared with other methods for RBs aggregation measurement, the electrical impedance spectroscopy method is much more suitable for the non-invasive measurements, as the electrical signal easily penetrates blood and tissues with quite fast frequencies, especially for the “point of care” devices. Therefore, some researchers have investigated the relationship between the electrical impedance/conductivity and RBC aggregation based on single-frequency electrical impedance measurements [30,31,32]. However, the previous impedance/conductivity measurements of RBC aggregation have the problem to distinguish the aggregation from the blood flow factors (RBC orientation, deformation and thickness of EDL). Additionally, impedance/conductivity-based single-frequency electrical impedance spectroscopy measurements are frequency dependent [28,29], and it is difficult to determine the appropriate frequency for each application. Moreover, there is still less of the quantitative calculation of the relationship between RBC aggregation and electrical impedance spectroscopy. Therefore, it is of great significance to develop a RBC aggregation measurement method that can overcome the blood flow factors (RBC orientation, deformation, thickness of EDL) to better evaluate RBC aggregation accurately. In our previous research, RBC aggregation during blood coagulation has been measured [13]. The influence of blood coagulation on RBC aggregability was investigated. However, even though there is no blood coagulation, the normal blood impedance parameters (impedance, conductivity, relaxation frequency) still differ with pulsatile flow. Why they are different, how to measure RBC aggregation in normal blood (non-coagulation), and how to quantitatively evaluate the influence of RBC aggregation on blood electrical impedance performance are still unsolved problems. 

In this study, RBC aggregation in normal blood was measured by multiple-frequency electrical impedance spectroscopy using the pulsatile blood flow. The influence of RBC aggregation on blood electrical impedance performance (impedance, conductivity, relaxation frequency) is investigated. The relaxation frequency *f*_c_ from the multiple-frequency electrical impedance spectroscopy Nyquist-plot is utilized to quantitatively investigate RBC aggregation. Additionally, the modified Hanai equation is proposed to quantitatively analyze the influence of RBC aggregation together with the RBC orientation, deformation, and thickness of EDL. This study brings new insights for the development of bio-sensors and bio-electronics for on-line RBC aggregation measurement technology by multiple-frequency electrical impedance spectroscopy.

## 2. Materials and Methods

### 2.1. Blood Samples

Both porcine and bovine blood were utilized in view of the ethical problem of using human blood and the large amount needed. In order to stop blood coagulation, trisodium citrate solution was added to the blood samples (volume fraction: blood/trisodium citrate solution = 9:1), immediately after the blood samples were withdrawn from porcine and bovine subjects. The animal blood was purchased from a slaughterhouse the morning before the experiments. The blood samples were then immediately transferred to the experimental room. The hematocrit of the porcine and bovine blood were around Hct = 41%, which was obtained by centrifugation for 5 min under a rotation speed of 20,000 r/min.

### 2.2. *Experimental Condition*

**Figure 1:**
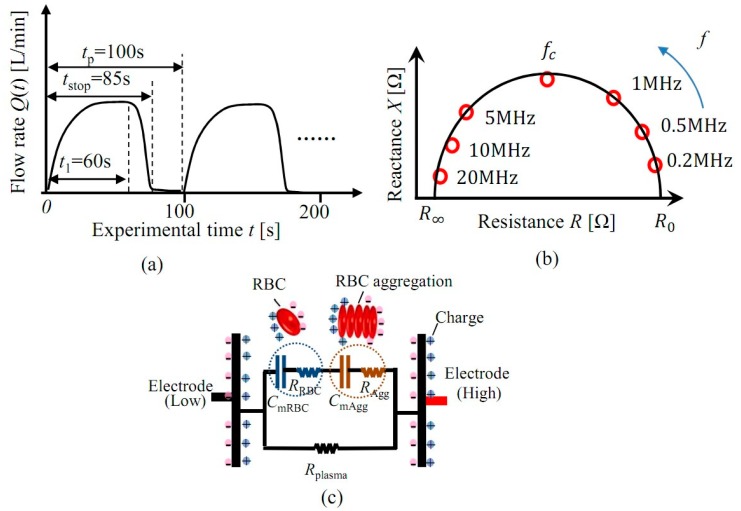
Experimental condition: (**a**) Pulsatile blood flow, (**b**) Nyquist plot, (**c**) Equivalent circuit model.

According to previous research, porcine blood easily generates RBC aggregates, whereas bovine blood does not. Hence, in this study, porcine blood is utilized for the RBC aggregation measurements, and bovine blood is for comparison. RBC aggregation is reversible, which means that aggregates can be separated by flow force. To clearly investigate the dielectric response of RBC aggregation, pulsatile blood flow is adopted by altering the rotation speed of the centrifugal pump with a pulsatile period of *t*_p_ = 100 s, as shown in Figure 1a. Flow rate *Q*(*t*) increases until *Q*_max_(*t*) = 2.70 L/min during *t*_1_ = 60 s, and after that it is suddenly decreased and maintained at *Q*(*t*) = 0 L/min after the time *t*_stop_ = 85 s. The total volume of the blood in the blood circulation system is around *V*_0_ = 700 mL. After that, the blood flow repeats this pulsatile pattern. The shear rate of blood inside the tube could be obtained by the following equation:

(1) $$\gamma_{m}=\frac{64Q(t)}{\pi d_{1}^{3}}$$

where *γ*_m_ is the mean shear rate of blood in the tube; *d*_1_ is the internal diameter of the used blood tube. Under the condition of the maximum flow rate, the mean shear rate is around 535 S^−1^, which is high enough to separate the RBC aggregates [22].

### 2.3. *Experimental Condition*

In this study, the relaxation frequency *f*c is utilized to evaluate the RBC aggregation, which is obtained from the Nyquist-plot fitted by the real part of impedance Z* (resistance *R*) and the image part of impedance Z* (reactance *X*), as shown in Figure 1b. The relaxation frequency *f*_c_ is obtained from the frequency of the highest point in Figure 1b. In porcine blood, RBCs always meet together to form a “rouleaux” aggregation, which changes the *f*_c_ of the blood. That is the reason why in this study *f*_c_ is found to be more suitable for the measurement of RBC aggregation. According to the equivalent circuit model of blood (shown in Figure 1c) and series-parallel circuit theory, which consists of plasma resistance *R*_plasma_, RBC resistance *R*_RBC_, RBC membrane capacitance *C*_mRBC_, RBC aggregation resistance *R*_Agg_ and RBC aggregate membrane capacitance *C*_mAgg_, the relaxation frequency *f*_c_ of blood is achieved by the following equation:

(2) $$f_{c}=\frac{1}{2\pi \cdot \left(\frac{R_{\mathrm{plasma}}\left(R_{\mathrm{RBC}}+R_{\mathrm{Agg}}\right)}{R_{\mathrm{plasma}}+R_{\mathrm{RBC}}+R_{\mathrm{Agg}}}+R_{\mathrm{plasma}}\right)\cdot (\frac{C_{\mathrm{mRBC}}C_{\mathrm{mAgg}}}{C_{\mathrm{mRBC}}+C_{\mathrm{mAgg}}})}$$

The value of relaxation frequency *f*_c_ is influenced by *R*_plasma_, *R*_RBC_, *C*_mRBC_, *R*_Agg_ and *C*_mAgg_, and it means that relaxation frequency *f*_c_ is much more sensitive to blood electrical characteristics change induced by RBC aggregation, compared with previous research based on impedance.

## 3. Results

### 3.1. Experimental Setup

**Figure 2:**
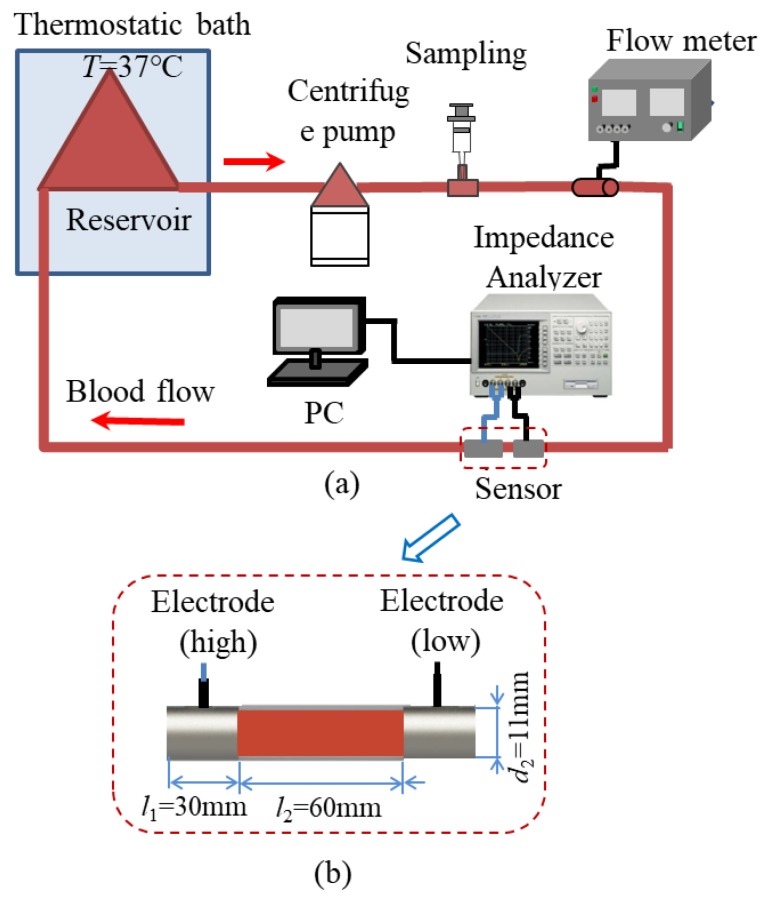
Experimental setup: (**a**) Blood circulation system, (**b**) Sensor.

Figure 2a shows the blood circulation system for the RBC aggregation experiments. A blood reservoir (Senko Medical Instrument Mfg. Co., Ltd., Kasukabe, Japan) is utilized to store the fresh blood, and it is kept inside a thermostatic bath at *T* = 37 ℃. A centrifugal pump (HCF-MP23, Mara, Kasukabe, Japan) is used to make the blood flow inside the blood tube. Different flow rates are obtained by controlling the rotation speed of the centrifugal pump. The flow rate is measured by a flow meter (T106, Transonic Systems Inc., Newyork, NY, USA). Two stainless steel rings with the length of *l*_1_ = 30 mm are applied as the sensor to measure the impedance of blood, and their sizes are illustrated in Figure 2b. The impedance signal is then obtained by an IM7581 impedance analyzer (Hoiki Company, Nagano, Japan) and analyzed by the personal computer (PC). The internal diameter of the blood tube used is around *d*_1_ = 9.5 mm, and the external diameter is around *d*_2_ = 11 mm. All of the blood tube and reservoir are coated with heparin to avoid coagulation. 

### 3.2. Impedance of the Pulsatile Blood Flow

**Figure 3:**
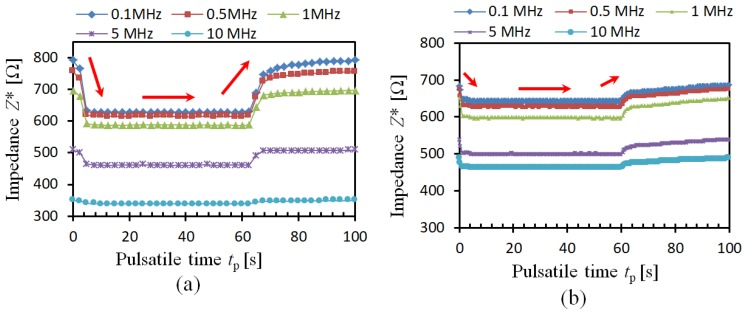
Experimental impedance of blood: (**a**) Porcine blood, (**b**) Bovine blood.

Previous electrical impedance spectroscopy studies on RBC aggregation measurements are all based on impedance/conductivity, which is frequency-dependent and not sufficient to distinguish the influence of RBC aggregation and blood flow factors (RBC orientation, deformation, thickness of EDL). Figure 3a illustrates the impedance performance of porcine blood with pulsatile flow during the experiments. It is observed that the impedance of porcine blood Z*(*t*) changes with the measurement frequency. In the case that the frequency is *f* = 0.1 MHz, at the beginning, the impedance of porcine blood Z*(*t*) is around *Z*_0_*(*t*) = 790.4 Ω. Then, the impedance of porcine blood Z*(*t*) decreases as flow rate *Q*(*t*) increases, and then the impedance remains almost constant around *Z*_s_*(*t*) = 626.4 Ω. In the case that the flow rate *Q*(*t*) falls after the time *t*_p_ = 60 s, the impedance of porcine blood Z*(*t*) goes up gradually to the original value *Z*_0_*(*t*) = 790.4 Ω. The impedance change shows the pulsatile regular pattern, which is thought to be caused by the pulsatile flow rate. Additionally, the impedance Z*(*t*) of porcine blood under different frequencies shows the same trend of variation, however, the rate of decrease of the impedance Z*(*t*) is becoming smaller in the case that frequency is higher. In the case that the frequency is *f* > 10 MHz, the decreasing and increasing variation are difficult to observe. 

The impedance Z*(*t*) of bovine blood presents a similar decreasing and increasing trend as that of porcine blood, which is shown in Figure 3b. However, the rate of decrease of the impedance Z*(*t*) is much smaller than the impedance decrease of porcine blood. Usually, according to the previous studies, the rate of decrease *g*(*f*) is obtained from the ratio of the impedance of blood at the original time *t*_p_ = 0 s and the constant time *t*_p_ = 40 s:

(3) $$g(f)\;=\;\frac{Z_{0}^{*}(t)-Z_{s}^{*}(t)}{Z_{0}^{*}(t)}$$

where *Z*_0_*(*t*) is the impedance of blood at the original time *t*_p_ = 0 s under the frequency of *f* = 0.1 MHz; *Z*_s_*(*t*) is the impedance of blood at the time *t*_p_ = 40 s under the frequency of *f* = 0.1 MHz that the impedance is almost constant.

According to Equation (3), both the rate of decrease *g*(*f*) of porcine blood and bovine blood are positive values. In the case the frequency is higher, the difference of *g*(*f*) becomes smaller. For example, the *g*(*f*) of porcine blood under the frequency of *f* = 10 MHz is 6.51%, whereas that of bovine blood is 5.13%. Even though the rate of decrease *g*(*f*) of porcine blood is a little higher than that of bovine blood, the value changes with different measurement frequency and different blood samples. In other words, with the previously used rate of decrease *g*(*f*) is difficult to differentiate the influence of RBC aggregation and blood flow factors.

### 3.3. Conductivity of the Pulsatile Blood Flow

**Figure 4:**
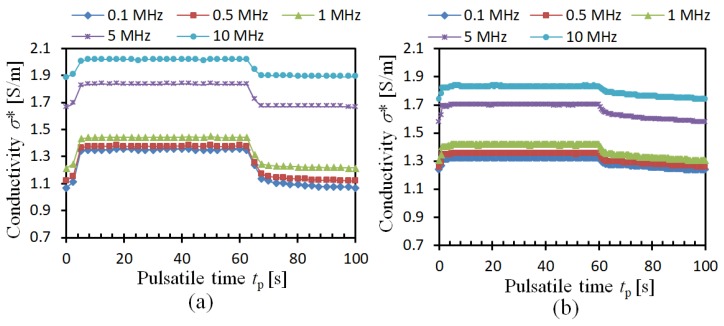
Experimental conductivity of blood: (**a**) Porcine blood, (**b**) Bovine blood.

Figure 4a illustrates the conductivity change of porcine blood under pulsatile flow. Different from the impedance performance, the conductivity σ^*^ of porcine blood increases with the flow rate *Q*(*t*). In the case that *f* = 0.1 MHz, the conductivity σ^*^ of porcine blood is σ^*^ = 1.07 S/m. It goes up to σ^*^ = 1.35 S/m, in the case that *Q*(*t*) is increasing. In the case that *Q*(*t*) decreases to zero, the σ^*^ falls down to the original value as well. The same trend is also found in bovine blood, which is shown in Figure 4b. It is obvious that the σ^*^ of both porcine blood and bovine blood are affected by the frequency and flow rate, which brings the difficulties to identify the RBC aggregation in these blood samples. It means that a much more effective parameter should be found to easily evaluate RBC aggregation.

### 3.4. Relaxation Frequency of Pulsatile Blood Flow

Compared with the previously used rate of decrease *g*(*f*) based on the impedance/conductivity, the proposed RBC aggregation parameter *R_ag_* based on relaxation frequency *f*_c_ from multiple-frequency electrical impedance spectroscopy measurements is confirmed to make it much more efficient and easier to quantitatively measure RBC aggregation. In this study, in order to investigate the influence of RBC aggregation on the dielectric characteristics of blood, and find a parameter to quantitatively evaluate the RBC aggregation, relaxation frequency *f*_c_ is measured during the pulsatile flow. 

**Figure 5:**
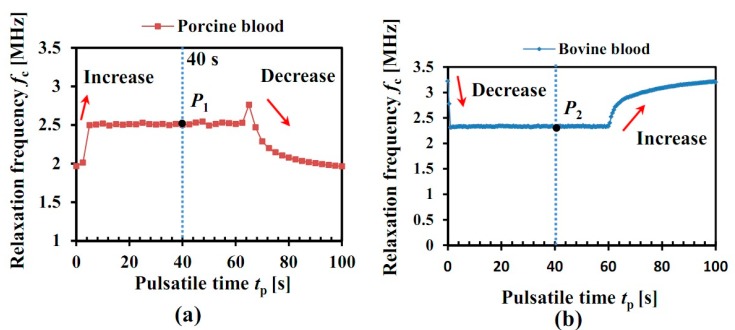
Experimental relaxation frequency of blood: (**a**) Porcine blood, (**b**) Bovine blood.

Figure 5a illustrates the relaxation frequency *f*_c_ of porcine blood under pulsatile flow rate *Q*(*t*). The *f*_c_ is obtained by the multiple-frequency electrical impedance spectroscopy and the Nyquist-plot introduced in Section 2. Hence, *f*_c_ is also called the multiple-frequency parameter. It is seen that, at the beginning, the *f*_c_ of porcine blood (red point) is around *f*_c_ = 1.96 MHz, and it increases quickly in the case that the flow rate *Q*(*t*) is increasing. Then, the *f*_c_ of porcine blood remains almost stable around *f*_c_ = 2.51 MHz until a gradual decrease is seen after the time *t*_p_ = 60 s. However, the *f*_c_ of bovine blood shows the opposite trend, seen in Figure 5b, it falls down sharply with the flow rate *Q*(*t*) from *f*_c_ = 3.22 MHz to *f*_c_ = 2.33 MHz, and then increases gradually in the case that the flow rate is stopped after the time *t*_p_ = 60 s. From the variation of *f*_c_, the behavior of porcine blood and bovine blood is quite different: one increases with flow rate, whereas the other one decreases. As mentioned above, the RBCs of porcine blood quite easily form the RBCs aggregates (“rouleaux”), whereas the RBCs of bovine blood do not. Here, the difference of *f*_c_ is thought to be caused by the RBC aggregation, which means that the RBC aggregation can be quantitatively investigated by *f*_c_. 

This study points out a novel method to measure RBC aggregation with pulsatile flow, and it will be quantitatively investigated in the discussion part. Equation (4) is proposed to quantitatively describe the RBC aggregation *R_ag_* based on the relaxation frequency. Unlike the previous research, the *f*_c_ of blood flow under both the static (*t*_p_ = 0 s) and flowing (*t*_p_ = 40 s) conditions are exploited:

(4) $$R_{\mathrm{ag}}\;=\;\frac{f_{c}^{}(40)-f_{c}^{}(0)}{f_{c}^{}(0)}$$

where *R_ag_* is the proposed RBCs aggregation parameter; *f*_c_(0) is the relaxation frequency at the time *t*_p_ = 0 s; *f*_c_(40) is the relaxation frequency at the time *t*_p_ = 40 s, when the relaxation frequency is stable.

According to the above equation, the RBC aggregation of porcine blood is *Ag* = 28.06%, while that of bovine blood is *R_ag_* = −27.64%. The positive value of *R_ag_* means the RBC aggregation, and the negative value of *R_ag_* is thought to be caused by the blood flow factors (RBC orientation, deformation, thickness of EDL) which will be further discussed in the discussion part. The difference in sign of the relaxation frequency for porcine blood and bovine blood is caused by the RBC aggregation, since porcine blood quite easily forms RBC aggregates while bovine blood does not. From the results, it is obvious that the proposed RBC parameter *R_ag_* based on the relaxation frequency is efficient to quantitatively describe the RBC aggregation and exclude the influence of blood flow factors.

## 4. Discussion

### 4.1. Modified Hanai Equation

In order to quantitatively investigate the influence of RBC aggregation based on relaxation frequency *f*_c_, and explain the relaxation frequency difference between porcine blood and bovine blood, the traditional Hanai equation for the electrical impedance calculation of inheterogeneous systems of dense colloids is modified from the view point of blood flow factors (RBC orientation, deformation, EDL thickness) and RBC aggregation. In this study, blood is simplified as a solution of RBCs and plasma, and the relaxation frequency *f*_c_ from modified Hanai equation is firstly utilized in the measurement of RBC aggregation during pulsatile flow. Since the blood flow inside the tube could be treated as Poiseuille flow, it indicates that the shear rate near the tube wall is higher than that near the tube center. This inhomogenerous distribution of shear rate means that the RBC aggregation is separated into a high shear rate region, and the RBC orientation, deformation, and thickness of EDL (blood flow factors) are also changed by the inhomogenerous shear rate.

In order to quantitatively calculate the influence of RBC aggregation during the pulsatile flow, the modified Hanai equation is obtained as follows:

(5) $$\begin{array}{l} \left(\frac{\varepsilon_{\mathrm{blood}}^{*}\left(r,t\right)-\varepsilon_{\mathrm{RBC}}^{*}\left(r,t\right)}{\varepsilon_{\mathrm{plasma}}^{*}\left(r,t\right)-\varepsilon_{\mathrm{RBC}}^{*}\left(r,t\right)}\right){\left(\frac{\varepsilon_{\mathrm{blood}}^{*}\left(r,t\right)}{\varepsilon_{\mathrm{plasma}}^{*}\left(r,t\right)}\right)}^{-C_{1}}{\left(\frac{\varepsilon_{\mathrm{blood}}^{*}\left(r,t\right)+A’\varepsilon_{\mathrm{RBC}}^{*}\left(r,t\right)}{\varepsilon_{\mathrm{plasma}}^{*}\left(r,t\right)+A’\varepsilon_{\mathrm{RBC}}^{*}\left(r,t\right)}\right)}^{-C_{2}}\cdot \\ {\left(\frac{\varepsilon_{\mathrm{blood}}^{*}\left(r,t\right)+B’\varepsilon_{\mathrm{RBC}}^{*}\left(r,t\right)}{\varepsilon_{\mathrm{plasma}}^{*}\left(r,t\right)+B’\varepsilon_{\mathrm{RBC}}^{*}\left(r,t\right)}\right)}^{-C_{3}}=1-H_{\mathrm{ct}} \end{array}$$

where ε^*^_blood_(*r*, *t*) is the permittivity of whole blood; ε^*^_RBC_(*r*, *t*) is the permittivity of RBC; ε^*^_plasma_ (*r*, *t*) is the permittivity of plasma; *H*_ct_ is the hematocrit of blood. 

**Figure 6:**
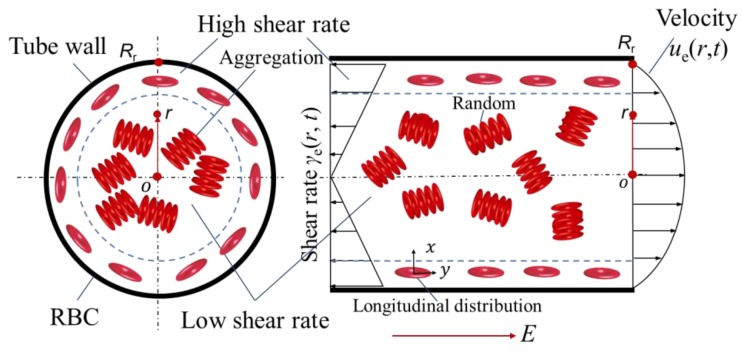
The inhomogeneous distribution of shear rate inside the blood tube.

It is known that RBCs are dish-like shape, so in this study, to simplify the calculation, RBCs are treated as elliptical particles. Due to the elliptical shape of RBCs, the orientation of RBCs in the tube will influence the dielectric characteristics of blood. It means that the RBCs near the tube wall (high shear rate) are oriented along the longitudinal direction (flow direction), RBCs aggregates are separated in the high shear rate region; the RBCs near the tube axis are in the low shear rate region, hence, they are easily form RBC aggregations and in a random direction, as is shown in Figure 6. Some parameters are obtained from previous studies [33,34].

The impedance of whole blood is achieved according to the above equations, as is shown in Equation (6). Moreover, the relaxation frequency *f*_c_ is then achieved by the Nyquist plot shown in Section 2:

(6) $$Z^{*}={\left(\frac{2\pi }{L}\int_{0}^{d_{1}/2}j\omega \varepsilon_{0}\varepsilon_{\mathrm{blood}}^{*}\left(r,t\right)rdr\right)}^{-1}$$

where *ε*_0_ is vacuum permittivity [31].

### 4.2. Shear Rate of the Pulsatile Flow

In this study, pulsatile flow is applied to separate the RBC aggregates, which means that the flow rate *Q*(*t*) changes with time (shown in Figure 1). Additionally, according to the theory of Poiseuille flow, the shear rate distribution of flow inside the tube is inhomogeneous, as is shown in Figure 6. Therefore, the shear rate *γ*_e_(*r*, *t*) is a function of the radial distance *r* and the time *t*. In order to quantitatively calcuate the influence of RBC aggregation, firstly, the velcocity *u*_e_(*r*, *t*) of blood flow inside the tube with circular cross section under the condition of pulsatile pressure gradient is obatained by the following equation [35]:

(7) $$u_{e}(r,t)=\frac{P_{e}}{i\omega \rho }\left[1-\frac{J_{0}\left(\alpha (r,t)yi^{3/2}\right)}{J_{0}\left(\alpha (r,t)i^{3/2}\right)}\right]e^{i\omega t}$$

(8) $$\alpha (r,t)=r\sqrt{\frac{\omega \rho }{\eta }}$$

where *α*(*r, t*) is the Womersley number which changes with the radial distance *r*, the angle speed *ω*, the density *ρ* of blood and the viscosity *η* of blood; *P*_e_ is the pressure coefficient; *r* is the radial distance to the tube center; *R*_r_ is the radius of blood tube; *y* is the distance ratio of the calculation point to the tube center, and the value is *y* = *r*/*R*_r_; i indicates the imaginary part; *J*_0_ is the zero order Bessel function. 

Therefore, the inhomogeneous shear rate *γ*_e_(*r*, *t*) of the blood inside the circular tube under the condition of pulsatile flow is achieved:

(9) $$\gamma_{e}(r,t)=\frac{du_{e}(r,t)}{dr}=\frac{P_{e}}{i\omega \rho }\left[\frac{\alpha (r,t)i^{3/2}J_{1}\left(\alpha (r,t)yi^{3/2}\right)}{J_{0}\left(\alpha (r,t)i^{3/2}\right)}\right]e^{i\omega t}$$

By Equation (8), the dynamic shear rate of the blood flow during pulsatile flow is quantitatively calculated. Then, it is utilized for the investigation of RBC aggregation during pulsatile flow.

### 4.3. Quantitative Calculation of RBC Aggregation

According to the experimental results in Figure 5, the relaxation frequencies *f*_c_ of porcine blood and bovine blood during pulsatile flow show the oppsite trend. In this study, the different behaviors are thought to be caused by the RBC aggregation. Due to the influence of shear rate *γ*_e_(*r*, *t*), the RBCs inside the blood tube show different behaviors: the RBC orientation, deformation, and the thickness of EDL are changed in the high shear rate region; the RBCs in the low shear rate region quite easily form RBC aggregates, as is shown in Figure 6. In the case that the real pulsatile flow rate is input into the above equations, and the modified Hanai equation (Equation (5)) is available for the quantitative calcuation of the influence of RBCs on *f*_c_.

**Figure 7:**
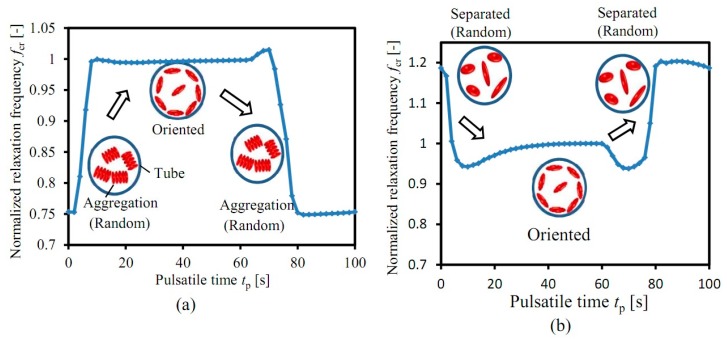
Calculation results based on the modified Hanai equation: (**a**) Porcine blood, (**b**) Bovine blood.

Figure 7 illustrates the calculation results of the influence of RBC aggregation on the *f*_c_ of blood based on the modified Hanai equation. It is seen in Figure 7a that, at the beginning, the flow rate *Q*(*t*) of porcine blood inside the tube is quite small, and so is the shear rate *γ*_e_(*r*, *t*). Hence, the RBCs quite easily form RBC aggregates. Here, to simplify the calculation, we assume that all of the RBCs are aggregated as the “rouleaux” structure, which is treated as a big particle with the same diameters *D*_y_ and *D*_z_ as a single RBC, and only the diameter in the *x* direction *D*_x_ changes. Moreover, the orientation of the RBC aggregation is random, so the the deformation and the thickness of RBCs remains stable. Then, with the increasing flow rate *Q*(*t*), the shear rate *γ*_e_(*r*, *t*) shows an inhomogeneous distribution. In the high shear rate region, the high shear rate separates the RBC aggregations into single RBCs. Additionally, the separated RBCs are oriented along the longitudinal direction, and their shape and thickness are deformed by the generated shear force. The normalized relaxation frequecy *f*_cr_ of porcine blood goes up quickly from *f*_cr_ = 0.75. After that, the normalized relaxation frequency *f*_cr_ keeps around *f*_cr_ = 1 in the case that all of the “rouleaux” aggregations of RBCs are separated and all of the RBCs are oriented. In the case that the pusatile time *t*_p_ > 60 s, the flow rate *Q*(*t*) is stopped, the shear rate *γ*_e_(*r*, *t*) becomes smaller, and then, the RBCs gather together again to form a “rouleaux” aggregation, and their shape and the thickness of the electrical double layer are restored to the orginal value. Therefore, the normalized relaxation frequency *f*_cr_ falls quickly from *f*_cr_ = 1 to *f*_cr_ = 0.75. 

Since it is difficult for bovine blood to form RBC aggregates, only the blood flow factors (RBC orientation, deformation, thickness of EDL) are changed with shear rate during the calculation based on the modified Hanai equation (no RBC aggregation). It is shown in Figure 7b that the normalized frequency *f*_cr_ of bovine blood presents the opposite behavior as that of porcine blood: it decreases quickly in the case that flow rate is increasing, then remains almost stable, and finally goes up quickly in the case that the flow rate is stopped. The relaxation frequency variation with the degree of RBCs aggreation has been studied in our previous work [13].

From the comparison of porcine blood and bovine blood, it could be found that the RBC aggregation greatly influences the relaxation frequency of blood. In other words, by measuring the relaxation frequency variation of blood during the pulsatile flow, the RBC aggregation is obtained quantitatively. 

## 5. Conclusions

In this study, the influence of RBC aggregation on the dielectric properties of normal blood (non-coagulation) during pulsatile flow has been quantitatively investigated. Different experiments with both porcine blood and bovine blood have been carried out under pulsatile flow. The experimental data indicate that the impedances Z*(*t*) of both the porcine blood and bovine blood decrease, while the blood flow rate *Q*(*t*) increases. However, the relaxation frequency *f*_c_ of porcine blood shows opposite behavior with that of bovine blood: it goes up from *f*_c_ = 1.96 MHz to *f*_c_ = 2.51 MHz, and then falls quickly in the case that blood flow is stopped; the *f*_c_ of bovine blood decreases at the beginning from *f*_c_ = 3.22 MHz to *f*_c_ = 2.33 MHz, and then increases quickly in the case that blood flow is stopped. The modified Hanai equation is proposed to quantitatively calculate the difference of the relaxation frequency. The results indicate that RBC aggregation is responsible for this phenomenon. The RBC aggregation parameter *R_ag_* is proposed to describe the RBC aggregation. This study confirms the feasibility of a novel RBC aggregation measurement method for CVDs based on the relaxation frequency *f*_c_ with pulsatile flow, which is effective to exclude the influence of blood flow factors (RBC orientation, deformation, thickness of electrical double layer).
